# Uric acid-based ratios for predicting renal failure in Chinese IgA nephropathy patients

**DOI:** 10.7150/ijms.85430

**Published:** 2023-09-25

**Authors:** Aiya Qin, Dandan Yang, Siqing Wang, Lingqiu Dong, Jiaxing Tan, Yi Tang, Wei Qin

**Affiliations:** Division of Nephrology, Department of Medicine, West China Hospital, Sichuan University, Chengdu, Sichuan, China.

**Keywords:** IgA nephropathy, IgAN, renal survival, uric acid to albumin ratio, UAR index

## Abstract

**Objective:** The uric acid/albumin ratio (UAR), a novel, simple, and compositive laboratory biomarker, has recently attracted attention for predicting disease prediction and disease prognosis. However, whether uric acid-related biomarkers (especially UAR) could serve as prognostic indicator for IgAN is unclear.

**Methods**: In this retrospective cohort study, biopsy-confirmed IgAN patients from 2009 to 2017 from West China Hospital were evaluated. The optimal cutoff value of UAR for renal outcome was defined using the Youden index by the area under receiver operating characteristic curve (AUC). The patients were then categorized into the high UAR group and the low UAR group. Renal endpoints were defined as progression to ESRD, eGFR decreased ≥50% of the baseline level, or initiation of renal replacement treatment. Kaplan‒Meier survival analysis and Cox regression analysis were used to identify factors influencing IgAN outcomes.

**Results:** A total of 1143 patients with a median age of 33.0 (26.0-42.0) (44.2% men) were included in the study. The best cut-off UAR concerned with renal survival was determined to be 9.94 with a specificity of 77.5% and a sensitivity of 61.5% (J, 0.390; AUC, 0.750). Then, the patients were divided into two groups labelled as low and high UAR ratios (≥ 9.94 and <9.94, respectively). More severe clinical manifestations and pathological lesions were observed in the high UAR group. Multivariate Cox regression analysis after adjusted for important clinicopathological parameters manifested that a high UAR was an independent prognostic biomarker for IgAN. (*p* = 0.036, HR =2.56, 95% CI: 1.07-6.16).

**Conclusion:** UAR might be a novel predictor for renal progression and contribute to targeted management.

## Introduction

IgA nephropathy (IgAN), featured by histopathological criteria of IgA deposits in the mesangium of the renal, is one of the most widespread type of primary glomerulonephritis worldwide 1. Nevertheless, the pathomechanism remains intricate and uncertain. The interactions of genes, immunity and the environment contribute to the variability of IgAN clinical manifestation 2. Approximately 30-40% of IgAN patients will develop into end-stage renal disease (ESRD) within 20 to 25 years from the onset of disease, which is associated with a tremendous economic burden, and the long-term prognosis remains relatively poor 3, 4. Consequently, early dentification of novel prognostic factors and taking corresponding measures is crucial to decelerate its progression towards ESRD.

As the final product of purine metabolism, uric acid (UA) is associated with oxidative stress, inflammatory response and endothelial dysfunction 5. To date, hyperuricemia, which is presented as elevated serum uric acid level and associated with metabolic derangement, has attracted widespread attention as a reliable prognostic indicator for IgAN progression 6, 7, and may contributes to renal injury through the activation of Wnt5a/Ror2 or the NADPH/ROS/ERk1/2 signaling pathway 8. Albumin (ALB), synthesized by the liver, is a negative acute phase reactant whose level in the blood decreases during malnutrition and inflammation 9, 10. Emerging studies have suggested that hypoalbuminemia is associated with poor renal progression 11, 12. In comparison, UAR is a novel composite index that is a combination of nutrition, inflammation and metabolic syndrome. It has attracted increasing attention for the prediction of contrast-induced nephropathy 13, the degree of coronary artery disease in non-ST segment elevation myocardial infarction patients 14, and for disease prognosis, such as acute kidney injury 15, 16 and ST-elevation myocardial infarction 17. However, no data exist regarding the association between UAR and renal outcome in IgAN patients. Thus, we set out this retrospective study in an effort to identify whether uric acid based markers (especially UAR) may help predict disease progression in Chinese IgAN patients.

## Method

### Patients

We evaluated IgAN patients aged ≥ 14 years old who underwent renal biopsies in West China Hospital between 2009 and 2017. Patients with renal biopsies containing fewer than eight glomeruli, no available MEST-C scores, renal failure at the time of kidney biopsy, follow-up less than 12 months, or secondary causes of mesangial IgA deposits (such as Henoch-Schonlein purpura, liver disease and systemic lupus erythematosus) or missing clinical data were excluded. The study was approved by the Ethics Committee of West China Hospital, Sichuan University (No. 2019-33). Written informed consent was obtained from all patients when recruited.

### Data

Medical records of each patient were carefully reviewed. The clinical data were collected which included albumin (ALB), hemoglobin (Hb), serum uric acid (UA), total cholesterol (TC), serum creatinine (Cr), estimated glomerular filtration rate (eGFR), urinary red blood cell counts (URBC), 24-h urinary total protein (24 h-UTP) and other laboratory indexes. The UAR was obtained by dividing the serum uric acid level by the plasma albumin level on admission as previous described 18. Oxford MEST classification was performed to grade the pathological lesions by at least two experienced pathologists at West China Hospital 3. Kidney biopsy samples were evaluated by light, immunofluorescence and electron microscopy.

### Treatment and Study endpoints

Treatment modalities were determined by clinical experience based on KDIGO guidelines and the patient's informed preference, which included adequate doses of renin-angiotensin-aldosterone system blockers, the standard-dose glucocorticoid regimen and other immunosuppressants. It is worth noting that the latter two were collectively defined as immunosuppressive therapy (IST). The studied outcome was defined as a composite outcome of a 50% decrease in eGFR from baseline or the presence of ESRD (eGFR < 15 mL/min/1.73 m^2^) or initiation of renal replacement treatment.

### Statistical Analysis

Continuous variables are expressed as mean ± s.d. or median with range and compared using the Mann-Whitney U test or t test. Categorical variables are presented as numbers with ratios, which were compared using Chi-squared or Fisher's exact tests.

Kaplan‒Meier survival analysis and Cox regression models were performed to analyze independent factors affecting IgAN prognosis. ROC curve analyses were applied to appraise the performance of UAR and other clinical parameters. Time-dependent receiver operating characteristic (td-ROC) curve were applied to appraise the Prediction accuracy of UAR at different times. A two-tailed *p* value <0.05 was regarded as statistically significant. All statistical analyses were accomplished by using SPSS (version 26.0; IBM Corp., NY, USA) and R (version 4.1.3).

## Results

### Study population

**Figure 1:**
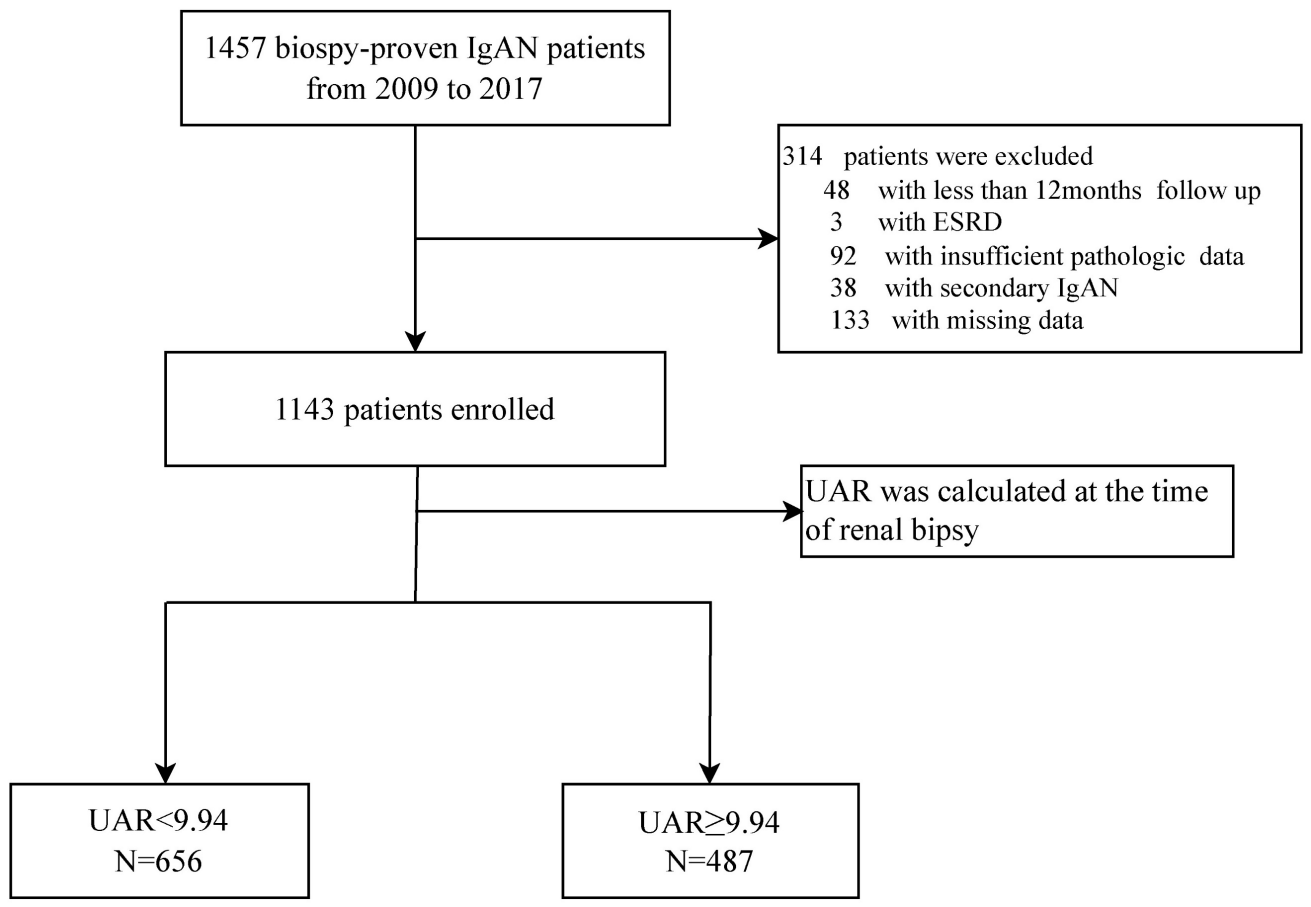
Flowchart of study participants.

**Figure 2:**
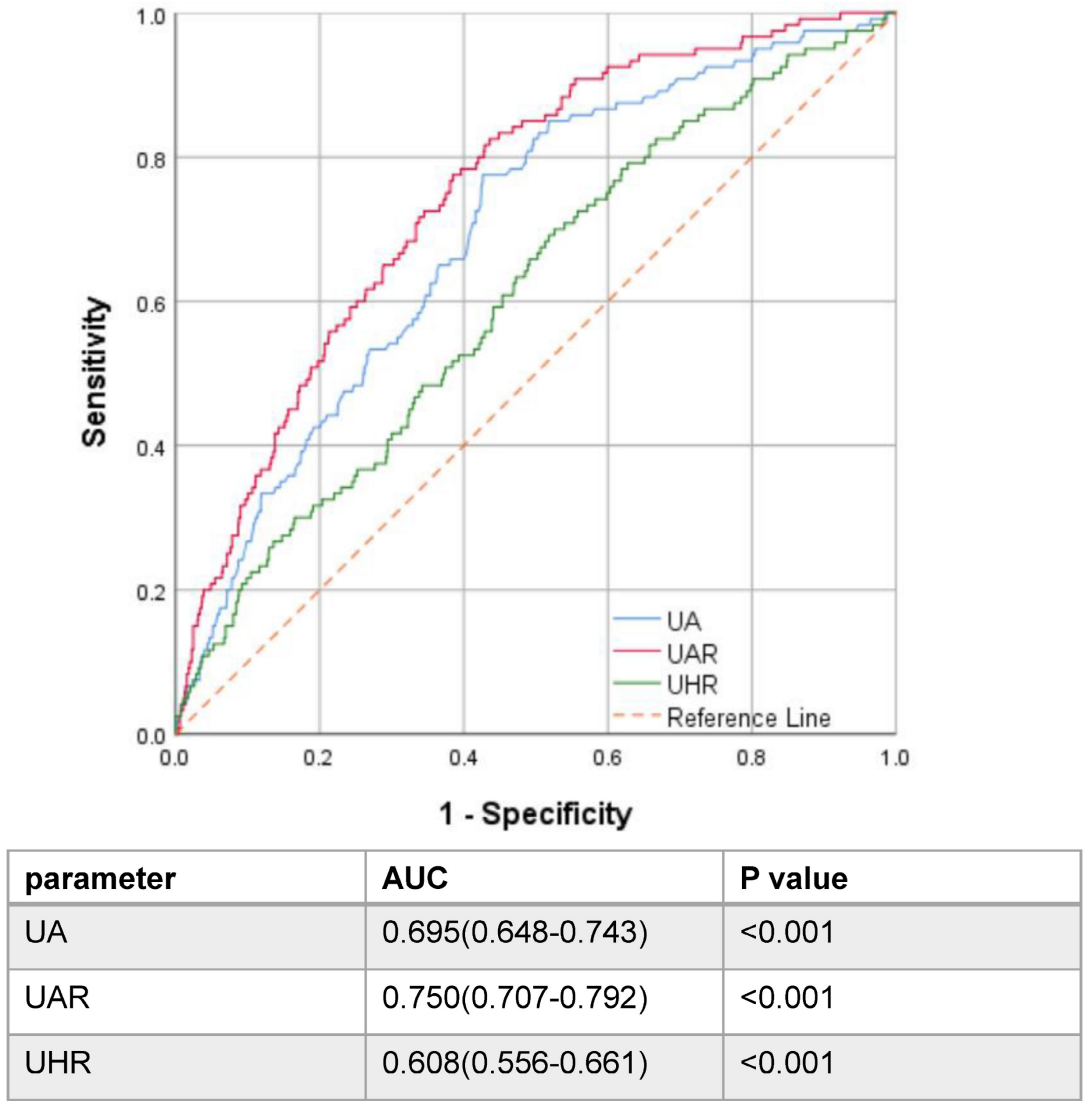
Receiver operating characteristic curve of uric acid-related parameters.

A flowchart of participants is shown in **Figure 1**. A total of 1457 pathological confirmed IgAN participants were initial identified and reviewed. Among them, 314 patients were excluded, remaining 1143 patients finally involved in this retrospective study. The patients with a median age of 33.0 (26.0-42.0) years old were followed up for a median of 56.3 (37.5-78.6) months. Notably, 120 patients progressed to composite end points, accounting for 10.5% of the total number. To assess the ability of UAR and other significant recently proposed uric acid related predictors by reviewing the literature to predict renal prognosis, such as UA, ALB, HDL, and UA/HDL ratios, ROC curve analyses were performed. ROC curves indicated that the best cut-off values were 9.94 for UAR with an AUC of 0.750, giving 77.5% sensitivity and 61.5% specificity for predicting renal survival among patients; 374.65 for UA with an AUC of 0.695, giving 77.5% sensitivity and 57.4% specificity; and 247.28 for UHR with an AUC of 0.608, giving 70.0% sensitivity and 47.4% specificity. It was shown that UAR had the best predictive power compared with UHR and UA alone (**Figure 2**). Then, all 1143 recruited patients (505 males and 638 females) were categorized based on the UAR on admission (low UAR group: n=656; high UAR group: n=487), with an overall cut-off value UAR. Compared with low UAR group, higher prevalence rates of hypertension and higher proportions of males, and smokers, as well as higher BMI, Cr, TG, TC, UA, 24 h-UTP levels, and worse renal function, were observed in the high UAR group, with more detailed baseline features displayed in **Table 1.** Although the trend was not statistically significant, higher Hb levels were observed in the high UAR group. Apparently, compared with the low UAR group, more patients reached the composite end points in the high UAR group (*p*=0.001).

### Pathological features

Regarding pathological features, the high UAR group tended to have more severe histopathological lesions (M, S, T). No significant differences were shown in immunofluorescence staining for IgG, IgM, C3, C4 and C1q between the two groups. As illustrated in **Table 2**, logistic regression revealed that there was a positive correlation between UAR and the Oxford classification. There was statistical significance in M_1_, S_1_, and T_1-2_ lesions, with odds ratios of 4.56 (2.19-9.46, *p* < 0.001), 2.29 (1.48-3.55, *p* < 0.001), and 8.90 (5.94-13.34, *p* < 0.001), respectively.

### Higher UAR is associated with worse renal survival in Chinese IgAN patients

**Figure 3:**
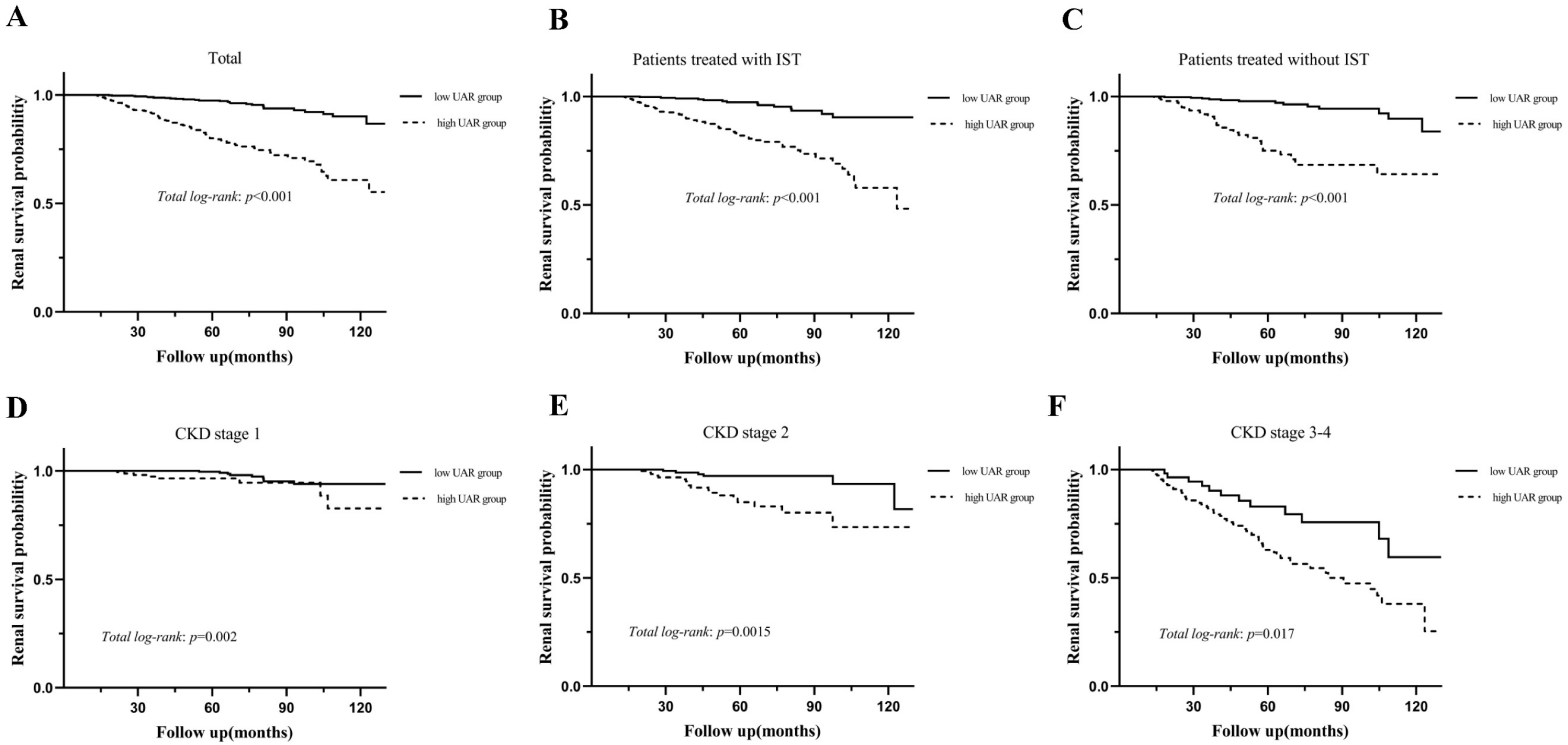
Kaplan-Meier analysis for the probability of renal endpoints grouped by treatment modalities and CKD stages. Abbreviations: IST, immunosuppressive therapy.

**Figure 4:**
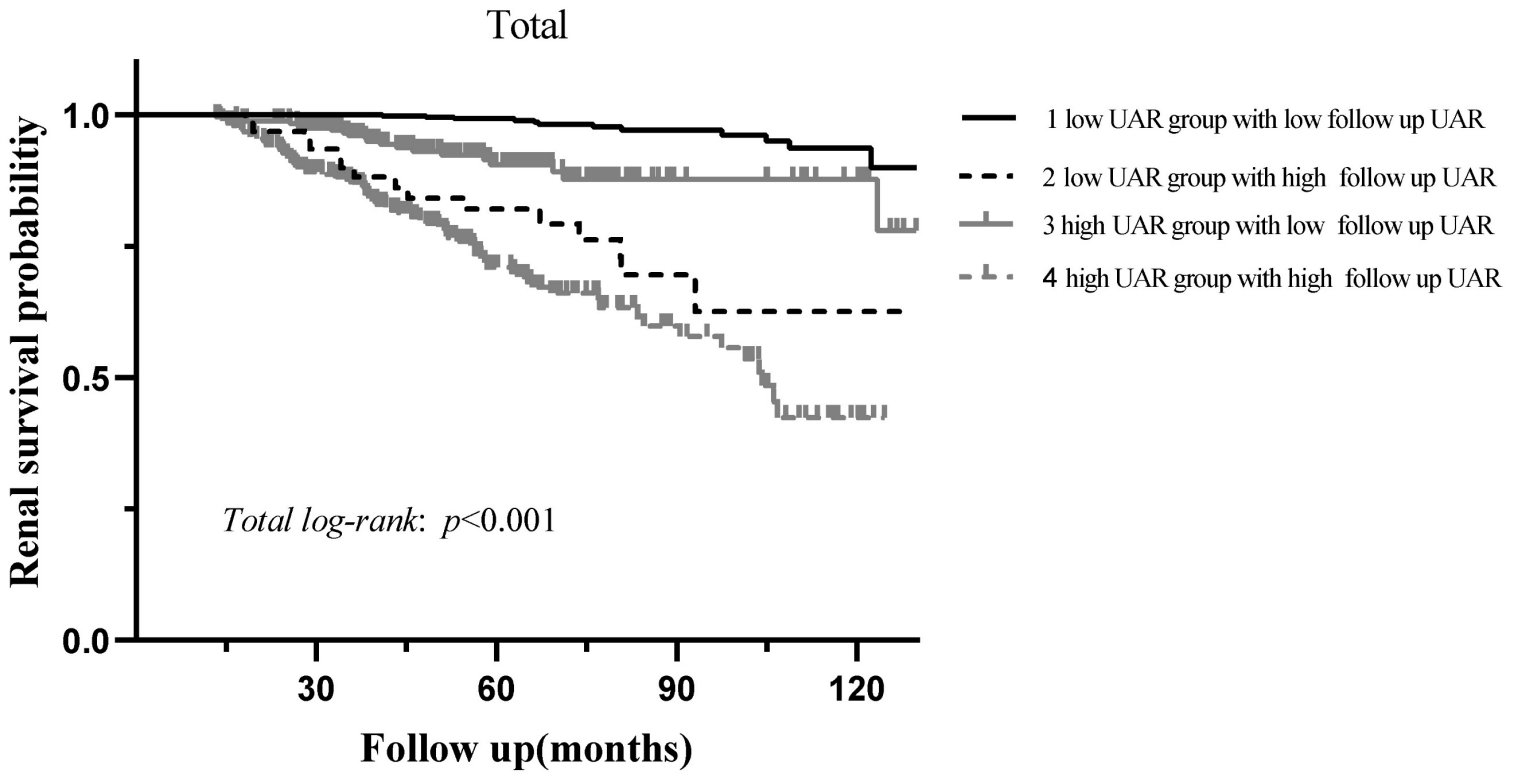
Kaplan-Meier analysis for renal survival grouped by baseline UAR levels and follow up UAR levels.

During the median 56.3 (37.5-78.6) months of follow-up, 120 (10.5%) patients progressed to the renal endpoints. Compared with the low UAR group patients, the high UAR group patients (93 of 487 patients, 19.1%) showed higher incidence of renal endpoints than those not (27 of 656 patients, 4.1%). As shown in **Figure 3A**, stratified by the UAR level, Kaplan-Meier survival curve for composite endpoints illustrated a significant difference between the two groups (*p* < 0.001). Subgroup analyses based on different treatment modalities (**Figure 3.B-C**) and CKD stages (**Figure 3.D-F**) were then carried out. Our results indicated that patients with high UAR levels ((UAR≥ 9.94) always had poor renal survival regardless of their treatment regimens and CKD stages (*p* < 0.001). Then, we detailed compared the value of UAR at the time of renal biopsy and the last follow-up UAR level for renal survival, which is presented in the **Figure 4**. The results indicated that the patients with persistently high UAR levels (UAR≥ 9.94) had the worst renal survival (*p* < 0.001).

### UAR and risk of progression to composite endpoints among Chinese IgAN patients

The results of Cox regression analysis were illustrated in **Table 3**. The univariate Cox analysis demonstrated that the UAR index was significantly related with a higher risk for composite endpoint [hazard ratio (HR) = 5.91, 95% CI 3.84-9.08, *p* <0.001]. In the further multivariate Cox analysis, model 1 (demographics + pathological features + UAR index), model 2 (demographics + clinical features+ UAR index) and model 3 (demographics + clinical+ pathological features+ UAR index) indicated that UAR was an independent factor of renal outcome (model 1: HR 3.78, 95% CI 2.36-6.04,* p*<0.001; model 2: HR 2.83, 95% CI 1.22-6.55, *p* =0.015; model 3: HR 2.56, 95% CI 1.07-6.16, *p* =0.036).

### Adding UAR to Baseline Model for Prediction of Outcomes

**Figure 5:**
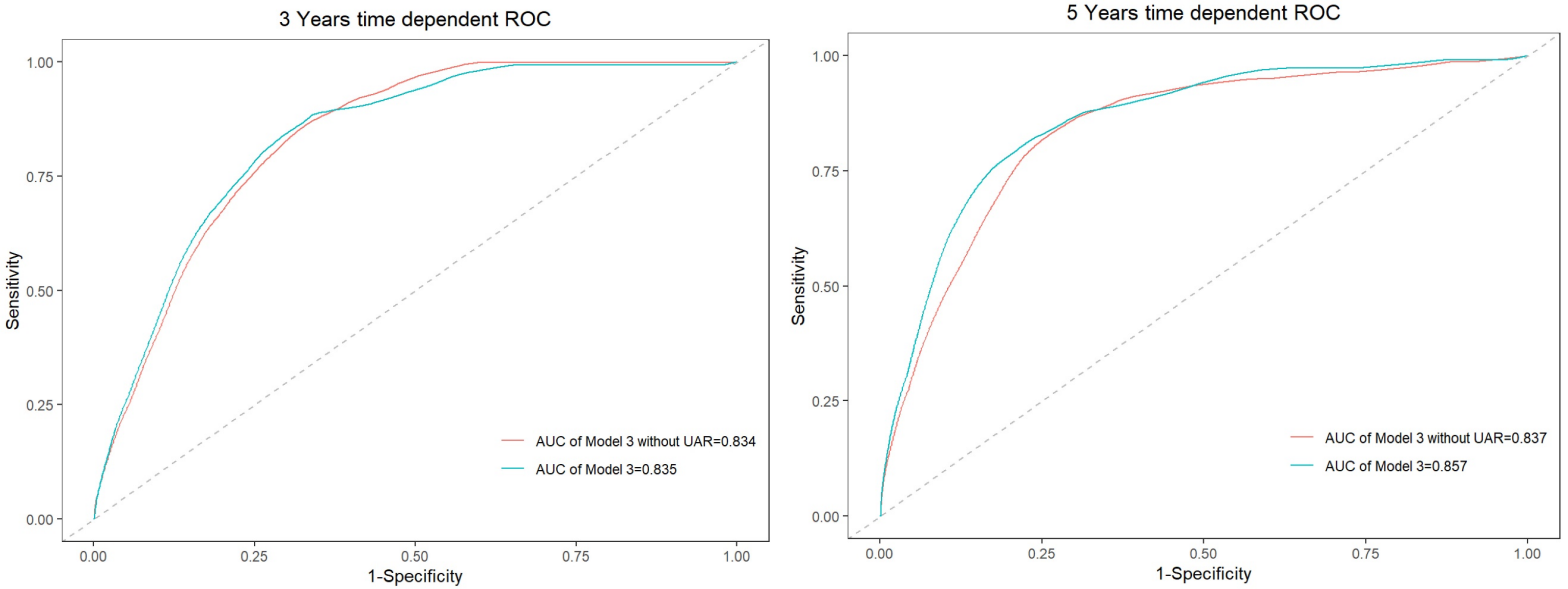
Time-dependent ROC curves for model 3 and the model without UAR.

Time-dependent receiver operating characteristics were used to better understand the prediction accuracy of UAR for composite endpoints, shown in **Figure 5**. When UAR was added to the model, the AUC value in evaluating 3-year and 5-year composite endpoints increased from 0.834 to 0.835 and 0.837 to 0.857, respectively. Thus, the predictive accuracy of the model incorporating traditional risk factors was significantly improved when the addition of UAR.

## Discussion

IgAN is the main disease leading to ESRD. Although the pathogenesis of IgAN remains complex and uncertain, it is well acknowledged that autoimmunity, inflammation, and nutrition play important roles in it 19. Identification of the risk factors and taking corresponding measures is crucial to decelerate its progression towards ESRD, reduce avoidable loss of life and the economic burden of disease.

Numerous researchers have examined the prognostic value of uric acid-based markers including UA, UAR and UHR. Compared with traditional parameters, such as UA, Alb, UHR, and UAR, the UAR showed the best optimal predictive performance, with the AUC of 0.750. A value greater than 0.7 indicates good predictive performance. By combining these two markers, a stronger and feasible predictor for renal progression was created. Then, the patients were divided into two groups labelled as low and high UAR by the optimal cut-off value (>9.94 and <9.94, respectively), in which the high UAR group had worse renal survival. The further analysis for the detailed follow-up UAR levels revealed that the patients with persistently high UAR levels (UAR≥ 9.94) had the worst renal survival. Our td-ROC also manifested that the prediction accuracy of the model 3 was significantly improved with the addition of UAR. To date, this is the first study to explore the association between UAR and renal progression in the Chinese IgAN population.

In line with our findings, many researches have manifested that UAR is related with kidney diseases. UAR has attracted increasing attention for the prediction of contrast-induced nephropathy 13, the degree of coronary artery disease in non-ST segment elevation myocardial infarction patients 14, and disease prognosis, such as acute kidney injury 15, 16 and ST-elevation myocardial infarction 17. In consequence, multiple studies with consistent results manifested that the UAR might be used in different clinical scenarios including improving risk stratification and predicting disease progression.

Emerging studies have suggested that systemic inflammatory response plays a vital role in the progression of IgAN 4, 19. Similarly, evidence also has shown that metabolic syndrome is relevant to IgAN progression 20, 21. Uric acid, the product of purine metabolism, is a white tasteless odorless crystalline, which is primarily cleared by the kidneys. Notably, abundant evidence has confirmed hyperuricemia, featured with an increase in serum uric acid, mediates renal impairment via a variety of mechanisms, which include oxidative stress, nitric oxide pathway alteration, insulin resistance (IR), fructose metabolism disorder, inflammatory activation, stimulation of the renin-angiotensin system, and endothelial dysfunction 6, 22, 23. The UAR, a valuable and compositive index that reflects the status of metabolic disturbance, nutrition and inflammation, possess the advantages of could be acquired timely and easily from initial serum biochemical analyses with low extra health cost, might be a novel predictor for renal progression and contribute to targeted management in the IgAN population.

Several limitations of this study are also worth mentioning. Firstly, this was a single-center cohort study with relatively short duration of follow-up. Secondly, due to its retrospective nature, here might lead to some selection bias. Moreover, regardless of the detailed data for urate-lowering therapy (ULT) were unavailable, by comparing the follow up UAR levels with the baseline, we found that patients with persistently high UAR levels (UAR≥ 9.94) had the worst renal survival. The results indicated IgAN patients with persistently high UAR levels had an increased risk of kidney failure events.

In the future, high-quality randomized controlled trials with longer follow-ups, larger sample sizes are required to determine whether early prevention and timely control of SUA levels (especially the use of the ULT) may delay kidney failure in patients with IgAN.

## Conclusion

Increased UAR may serve as a novel and reliable indicator for renal progression in Chinese IgAN.

## Figures and Tables

**Table 1:** Clinicopathological characteristics at the time of renal biopsy.

Parameters	Total N=1143	Low UAR group (UAR<9.94) N=656	High UAR group (UAR ≥ 9.94) N=487	*p* value
Age (year)	33.0(26.0-42.0)	31.5(26.0-40.0)	34.0(27.0-44.0)	0.004
Sex (male)	505(44.2)	215(32.8)	290(59.5)	<0.001
SBP (mmHg)	115(125(-138)	123.5(113.0-135.0)	128.0(117.0-142.0)	<0.001
DBP (mmHg)	81.0(74.0-90)	80.0(73.0-88.0)	84.0(76.0-93.0)	<0.001
BMI(Kg/m^2^)	22.7(20.0-25.4)	22.2(19.6-25.0)	23.4(20.7-26.1)	0.001
hypertension (%)	325(28.4)	129(19.7)	196(40.2)	<0.001
Smoking (%)	204(17.8)	71(10.8)	133(27.3)	<0.001
**CKD stages (%)**				<0.001
Stage 1	619(54.2)	449(68.4)	170(34.9)	
Stage 2	219(25.5)	152(23.2)	139(28.5)	
Stage 3	202(17.7)	50(7.6)	152(31.2)	
Stage 4	31(2.7)	5(0.8)	26(5.3)	
**Oxford Classification**				
M1 (%)	884(77.3)	485(73.9)	399(81.9)	0.002
E1 (%)	49(4.3)	22(3.4)	27(5.5)	0.077
S1 (%)	682(59.7)	368(56.1)	314(65.4)	0.005
T1/T2 (%)	225(19.7)	58(8.8)	167(34.3)	<0.001
C1/C2 (%)	245(21.4)	134(20.4)	111(22.8)	0.344
**Immunofluorescence**				
IgG (%)	81(7.1)	52(7.9)	29(6.0)	0.244
IgM (%)	438(38.3)	267(40.7)	171(35.1)	0.057
C3 (%)	924(80.8)	543(82.8)	381(78.2)	0.058
C4 (%)	31(2.7)	16(2.4)	15(3.1)	0.582
C1q (%)	88(7.7)	53(8.1)	35(7.2)	0.654
**Clinical**				
Cr (umol/L)	83.0(64.2-108.4)	71.0(59.1-89.5)	101.8(81.0-138.0)	<0.001
eGFR (mL/min/1.73m^2^)	93.9(66.3-117.6)	105.3(82.5-121.4)	72.3(49.8-101.2)	<0.001
ALB(g/L)	40.1(36.3-43.2)	41.3(38.5-44.3)	37.6(32.6-41.4)	<0.001
TG (mmol/L)	1.5(1.1-2.2)	1.3(0.9-1.9)	1.8(1.2-2.5)	<0.001
TC (mmol/L)	4.8(4.1-5.6)	4.6(3.9-5.3)	5.1(4.3-6.1)	<0.001
HDL (mmol/L)	1.4(1.1-1.7)	1.4(1.2-1.7)	1.3(1.0-1.7)	<0.001
24 h-UTP (g/d)	1.3(0.7-2.8)	1.0(0.6-2.0)	2.0(1.0-3.8)	<0.001
URBC (/HP)	18.0(6.0-59.0)	22.0(8.0-69.0)	14.0(5.0-50.0)	<0.001
Hb (g/L)	133.0(120.0-146.0)	132.0(120.0-144.0)	135.0(119.0-149.0)	0.108
UA (umol/L)	363.0(297.0-440.0)	314.5(265.0-358.0)	451.0(397.0-506.0)	<0.001
**Treatment** (%)				<0.001
SC	656(57.4)	323(49.2)	141(29.0)	
CS or/and IT	487(42.6)	333(50.8)	346(71.0)	
**Follow-up (m)**	56.3(37.5-78.6)	60.9(39.8-83.8)	51.4(35.5-71.0)	<0.001
**Outcome**				
Composite end point	120(10.5)	27(4.1)	93(19.1)	<0.001

**Abbreviations:** SBP, systolic blood pressure; DBP, diastolic blood pressure; BMI, body mass index; CKD, chronic kidney disease; M, mesangial proliferation; E, endocapillary proliferation; S, segmental glomerulosclerosis; T, tubular atrophy or interstitial fibrosis; C, crescents; IgG, immunoglobulin G; IgM, immunoglobulin M; C3, complement C3; C4, complement C4; C1q, complement C1q; Cr, serum creatinine; eGFR, estimated glomerular filtration rate; ALB, albumin; TG, serum triglycerides; TC, serum total cholesterol; HDL, high-density lipoprotein; URBC, urinary red blood cell counts; 24 h-UTP, 24 h urine total protein; Hb, hemoglobin UA, uric acid; SC, supportive care; CS, corticosteroids; IT, immunosuppressive therapy.

**Table 2:** Logistic model for the relationship between UAR and kidney pathologic lesion.

	OR	95%CI	*p* value
M	4.56	2.19-9.46	<0.001
E	1.45	0.64-3.30	0.379
S	2.29	1.48-3.55	<0.001
T1-2/T0	8.90	5.94-13.34	<0.001
C1-2/T0	1.38	0.90-2.13	0.141
Hypertension	2.86	1.95-4.20	<0.001
Smoking	1.89	1.23-2.91	0.004

**Abbreviations:** M, mesangial proliferation; E, endocapillary proliferation; S, segmental glomerulosclerosis; T, tubular atrophy or interstitial fibrosis; C, crescents.

**Table 3:** Multivariate Cox analyses for UAR associated with renal outcome.

Variable	Univariant		Model 1		Model 2		Model 3	
	HR (95% CI)	*p*	HR (95%CI)	*p*	HR (95%CI)	*p*	HR (95%CI)	*p*
UAR	5.91(3.84-9.08)	<0.001	3.78(2.36-6.04)	<0.001	2.83(1.22-6.55)	0.015	2.56(1.07-6.16)	0.036

Model 1 adjusted age, gender, Oxford classification of IgA (MEST-C scores). Model 2 adjusted age, gender, BMI, hypertension, smoking, CKD stages, proteinuria, URBC, Hb, total TG, TC, HDL, and treatments. Model 3 adjusted covariates in model 1 and model 2.
